# A Retrospective Study of Incidence and Predictors on Mother-to-Child Transmission of HIV among HIV-Exposed Infants in West Guji Zone, Southern Ethiopia

**DOI:** 10.1155/2022/2906490

**Published:** 2022-02-23

**Authors:** Girish Degavi, Boko Loka Safayi, Shiferaw Gelchu Adola, Biniyam Demisse, Takala Utura, Udessa Gemeda, Sarah Ezhil Kelna Edwin, Fitsum Demissie

**Affiliations:** ^1^School of Nursing and Midwifery, Institute of Health, Bule Hora University, Hageremaryam, Ethiopia; ^2^School of Nursing, College of Medicine and Health Science, Arbaminch University, Arbaminch, Ethiopia; ^3^School of Public Health, Institute of Health, Bule Hora University, Hageremaryam, Ethiopia; ^4^Department of Pharmacy, Institute of Health, Bule Hora University, Hageremaryam, Ethiopia

## Abstract

**Background:**

The transmission of HIV from mother to child among HIV-positive infants is estimated to be higher than 20%, despite the fact that antiretroviral treatment is available for antenatal mothers with HIV. In Ethiopia, the prevalence of HIV transmission from mother to child among infants aged one and a half years is estimated to be approximately 15.7 percent.

**Methods:**

A retrospective cohort analysis using a simple random sampling technique was incorporated among 422 HIV-exposed babies and their mothers who were randomly chosen and screened using OPD (outpatient card) from March 2019 to March 2021 in the general hospitals of West Guji zone, Oromia, Ethiopia. The data were coded and entered into EpiData version 4.6.1 and exported to SPSS version 23 for cleaning and analysis.

**Result:**

The study revealed that at the end of follow-up, 3.8% of the HIV-exposed infants were found to be HIV positive. Poor adherence of infant for CPT (AOR: 5.6; 95% CI: 1.010–27.24), father not enrolled to ART (AOR: 4.4; 95% CI: 1.187–15.724), age of infants at enrollment >6 weeks (AOR: 4.5; 95% CI: 1.102–16.1), mother's enrollment to PMTCT during labor and delivery or after (AOR: 6.84; 95% CI: 1.316–42.743), and mothers on the WHO clinical stage mild or advanced (AOR: 3.6; 95% CI: 1.146–16.842) was found to be the most important significant predictors of mother-to-child transmission of HIV.

**Conclusion:**

Several factors included in the study were the main predictors of mother-to-child transmission of HIV. The study concluded that there are some lacunae in the prevention of MTCT of HIV but that the incidence of MTCT of HIV was significantly lower in this part of the world.

## 1. Introduction

The first reports of AIDS in children were found nearly 18 months after the first case was found in the adult population which was reported in the year 1982. MTCT is the transmission of HIV from an HIV-positive mother to her child during birth and continues to be a threat up to the postnatal phase or the cessation of breastfeeding which accounts for more than 90% of HIV infections in infants. Exposed infants are infants born from mothers with positive serostatus of HIV and can be infected with HIV during pregnancy, labor, or through breastfeeding [1–3].

The first confirmed cases of HIV in Ethiopia were discovered in 1984, and the disease has since taken the lives of millions of people and left thousands of orphans. Mostly in the early 2000s, this led to the emergence of antiretroviral therapy (HAART) in infinite resource settings [3, 4].

The Ethiopian federal ministry of health (FMoH) implemented the PMTCT program in 2011 to provide effective interventions on MTCT of HIV during pregnancy, labor and delivery, and the breastfeeding period by providing ARV drugs for the mother and the baby. In addition, an option *B*+ approach which is immediate initiation of lifelong ART regardless of clinical stage and CD4 cell count for all HIV-infected pregnant and breastfeeding women had been implemented in 2013. The implementation of the global plan had a substantial impact, leading to a 60% reduction in new pediatric HIV infections [5].

Despite the fact that different governmental and nongovernmental organizations are struggling to retain HIV-exposed infants in care until the end of exposure, around 16% of HIV-exposed infants were infected with HIV in 2012 in 21 high-burden countries in sub-Saharan Africa, but still the burden of new HIV infections in children secondary to MTCT remains significantly high. Infants below the age of 18 months who had been withdrawn from breastfeeding for at least six weeks prior to data collection and who had been diagnosed with HIV were found to be positive by virological tests using nucleic acid testing (NAT) and quick HIV antibody testing which had also been used to detect HIV infection in the same age group [3, 6, 7].

In the middle of the 20^th^ century, only 50% of HIV-exposed infants were been tested, and it is believed that only 30% of the prenatally infected children were attached to ART services globally [7]. In 2016, only around half of these HIV-positive children underwent antiretroviral therapy [8]. According to the Ethiopian population health survey (EDHS) 2016, the number of children screened for HIV was relatively low, with about 22% in metros and just 5% in rural areas [9].

Pediatric HIV infection is a major public health concern that overwhelmingly affects children in poverty-trapped regions [10]. 1.7 million children under the age of 15 are among the 37.9 million people infected with HIV worldwide to date [11]. In 2018, about 9.4 percent of the 1.7 million new HIV infections worldwide were in children aged 0 to 14 years old, with 90 percent of the infected as a result of MTCT during pregnancy, labor, and birth, as well as during breastfeeding [3, 11–14].

With 25.7 million people living with HIV in 2018, Africa is the most affected country, responsible for nearly two-thirds of all new HIV infections worldwide [3, 12]. Ethiopia is one of the 21 high-burden nations, with an HIV prevalence of 0.96 percent, varying from less than 0.1 percent in Ethiopia's Somali region to 4.8 percent in the Gambella region [3, 11]. There are 610,335 people living with HIV/AIDS, with 13,488 people newly infected in 2018, 62 percent of whom are female [3].

Despite the fact that a variety of studies on the prevalence, predictors, and MTCT of HIV have been performed in various regions of Ethiopia, the majority of them have been known as single health institutions with small sample sizes. Furthermore, some researchers use a nonprobability sampling method and therefore do not take into account the breastfeeding interval after six months of age. The study tries to identify the relatedness of MTCT of HIV specifically while covering the period, which includes the breastfeeding time as well, which has not been explored in Ethiopia.

## 2. Methods

### 2.1. Study Setting and Period

The study was conducted in public general hospitals of the West Guji zone providing PMTCT service from March 2019 to March 2021. The study areas included Bule Hora General Hospital and Karcha General Hospital. A two-year institutional-based retrospective cohort study design was used on HIV-exposed infants and their mothers using the patient charts registered from March 2019 to March 2021 were included. As for the registration of HIV-exposed infants, follow-up of the selected general hospitals shows that there are 461 HIV-exposed infants registered in the two-year follow-up period.

### 2.2. Source Population

All HIV-exposed infants in all general hospitals of the West Guji zone registered were from March 2019 to March 2021.

### 2.3. Inclusion Criteria

All HIV-exposed infants who have undergone a deoxyribonucleic acid-polymerase chain reaction test (DNA-PCR) prior to 18 months of age were included. Children who had undergone rapid antibody tests done during follow-up six weeks after cessation of breastfeeding and who had been registered from March 2019 to March 2021 at the selected general hospitals of the West Guji zone were included in the study.

### 2.4. Exclusion Criteria

HIV-exposed infants enrolled in PMTCT service from March 2019 to March 2021 in the selected general hospitals of the West Guji zone who had not confirmed their HIV test results were excluded from the study.

### 2.5. Determination of Sample Size

For the first objective single proportion formula was made by considering 15.7% mother-to-child transmission rate of HIV among exposed infants on care and follow-up from a previous study [15, 16].

For the second objective using the double proportion formula, the sample size was calculated using Epi Info Version 7 Stat Calc by the assumption of 95% CI and 80% power with an exposed-to-unexposed ratio of 1 : 1 and using a 1.5 design effect, by considering variables like infant feeding practice, infant age at enrollment to PMTCT, residence, and antenatal care (ANC) follow-up as the major predictor variables. Moreover, infant feeding practice was considered as the independent predictor since it gives the maximum sample size (to reduce the role of chance), so *n* = 422.

### 2.6. Sampling Technique and Procedure

First, the public general hospitals of West Guji zone providing PMTCT service were listed. Then, from all the general hospitals in West Guji zone providing PMTCT service 60% of them (2 general hospitals) were selected by using the lottery method. The selected general hospitals that were included in the study were Bule Hora General Hospital and Karcha General Hospital. Then, the sample size for each selected general hospital was proportionally allocated according to the number of HIV-exposed infants registered for follow-up from March 2019 to March 2021. After that, medical records of pairs of exposed infants and their mothers were selected by using simple random sampling from the registration book of exposed infants' follow-up at the selected hospitals.

### 2.7. Data Processing and Analysis Procedure

The data were coded and entered into EpiData version 4.6.1 and was exported to SPSS version 23 for cleaning and analysis. Cleaning of data was made to check for any inconsistencies, errors in coding, missing values, out-of-range values, unexpected data or outliers, and inconsistencies were cross-checked with the data in hard copy and necessary correction measures were taken. Descriptive statics were presented through tables, frequencies, texts, mean, median, and figures. The HIV serostatus of the exposed infants at the age of 18 months or the last result after six weeks of cessation of breastfeeding was used to compute the incidence of MTCT of HIV among HIV-exposed infants. Bivariate and multivariate logistic regression analysis with a 95% confidence interval was employed to assess the association between the independent variable and the outcome variable. Variables with a *p* value less than 0.25 in bivariate analysis were entered into a multivariable logistic regression analysis to examine the main predictors of mother-to-child transmission of HIV. Variables that persisted to be associated with the outcome at *P* < 0.05 were used to declare whether the association between the outcome and independent variables exist.

## 3. Result

### 3.1. Sociodemographic Characteristics

**Figure 1:**
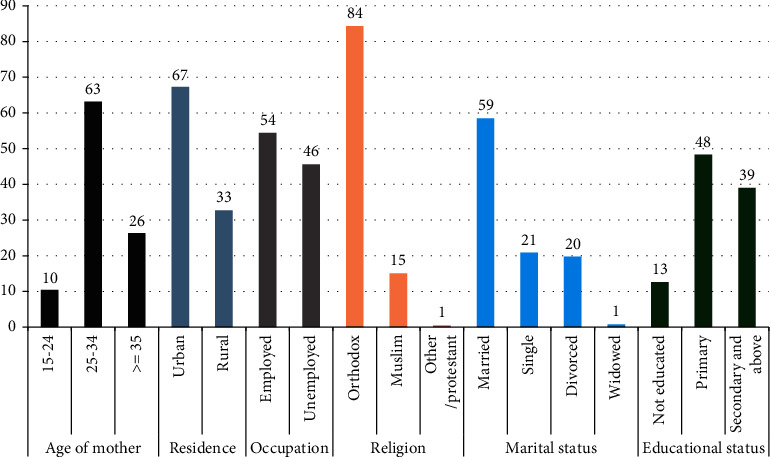
Sociodemographic characteristics of mothers enrolled in PMTCT care in selected public general hospitals of West Guji zone, Ethiopia, from March 2019 to March 2021.

**Figure 2:**
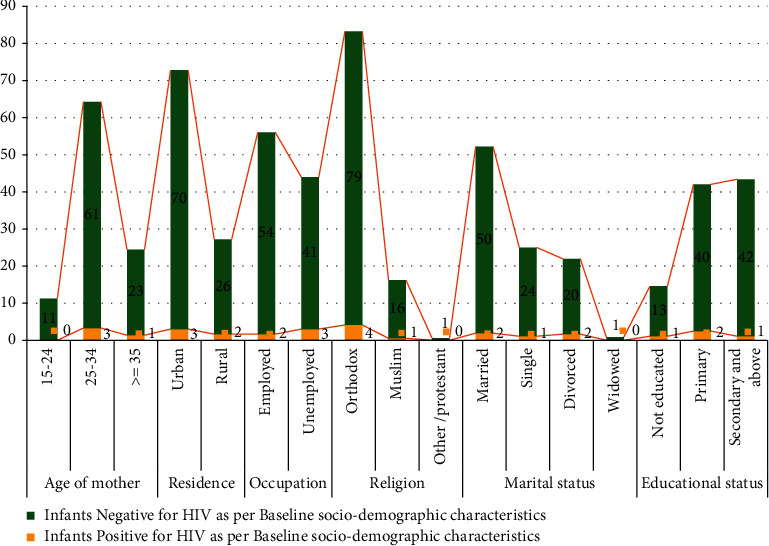
Sociodemographic characteristics of infants with HIV status as per baseline sociodemographic characteristics in selected public general hospitals of West Guji zone, Ethiopia, from March 2019 to March 2021.

The data from 462 HIV-exposed infants and their mothers enrolled in PMTCT care in selected public general hospitals of the West Guji zone, Ethiopia, from March 2019 to March 2021 were reviewed. Of these, 422 (91.4%) HIV-exposed infants and their mothers were included in the final analysis, and 34 (9.34%) were incomplete charts. The study included a two-year duration PMTCT data, registered from March 2019 to March 2021. The data were collected from two general hospitals. Most of the data were collected from Blue Hora Hospital. More than half of mothers were in the age group of 25–34 years, and 26% were ≥ 35 years. Almost 60% of women were married, and 21.0%, 20.0%, and 1.0% were single, divorced, and widowed, respectively. Almost half of the mothers had only elementary education, and half of the mothers were employed. 84% of study participants were followers of the orthodox religion (Figures 1 and 2).

### 3.2. Clinical Characteristics of the Mother

The data on maternal clinical conditions were collected at baseline and follow-up. More than 71% of mothers were working, ambulatory, and bedridden at baseline. A maximum number of mothers knew their HIV status before and after pregnancy while all mothers were enrolled to PMTCT. About 272 mothers were on HAART before pregnancy, above 60% had good ART adherence during pregnancy, and above 85% had fair adherence. Eighty-six mothers had a 200–350 cell/mm^3^ CD4 count which was quite significant. During pregnancy, around 39% of the mothers had ≥ 500 cells/mm^3^, respectively. The WHO clinical stage of the mothers at baseline was found to be more than 20%, and during pregnancy, most of them were on mild WHO clinical stages. Two hundred forty mothers had at least one opportunistic infection at baseline. Of those with OIs, participants were found to suffer from TB, diarrhea, cutaneous manifestations, and recurrent URTI for one month and above. Most of the mothers had disclosed their HIV status to others as well as to their husbands and relatives. A majority of the mothers had an undetectable viral load during pregnancy. Their nutritional status during pregnancy was significantly higher among samples with no malnutrition. Most of the mothers tested negative for syphilis tests during pregnancy (Table 1).

### 3.3. Obstetrics Characteristics of the Mother

The obstetric history of mothers stated maximum study participants were of gravid two, three, and above. Almost all, 93 percent of the mothers had ANC follow-up. Of these above 60% had ≥ 4 visits. Almost all of the mothers delivered at health institutions, among whom above 80% delivered through SVD. Nearly 64% of the mothers had postnatal follow-up after delivery of the index pregnancy.

### 3.4. Infant Characteristics of HIV-Exposed Infants and Incidence of MTCT of HIV

At the end of follow-up, 27 (6%) of HIV-exposed infants were confirmed to be HIV positive. Nearly 226 (53%) of the children were male. The birth weight of about 93% of the exposed infants was ≥ 2.5 kg with a mean of 2.84 kg and a SD of 0.412. The growth pattern of the majority of infants was normal. Almost all infants were on exclusive breastfeeding and none of them were on mixed feeding until 6 months of age. After six months of age, nearly 400 of the exposed infants were on breastfeeding and complementary feeding, only 6% stopped breastfeeding at six months of age. Most of the exposed infants (82%) were enrolled in PMTCT at the age of < = 6 weeks. All of the exposed infants received ARV prophylaxis and their adherence was significantly good, far, and poor, respectively. Above 60% of the fathers of these exposed infants were positive, and among those positive fathers, most of them were enrolled in ART.

The transmission rate was found more among males, and the majority of the infants who were delivered at home were HIV positive compared to infants delivered at health institutions. The transmission rate was also higher among infants who did not receive ARV prophylaxis at birth compared to those who received it. The transmission rate among rural residents was found to be comparatively more than in urban areas (Table 2).

### 3.5. Predictors of MTCT of HIV

The relationship between the independent variables and the incidence of MTCT of HIV was analyzed using a logistic regression model. In the bivariate logistic regression analysis, the independent variables mother's place of residence, occupation of mother, marital status, educational status of the mother, time of knowing HIV status of the mother, time of enrollment to PMTCT, disclosure status of the mother, viral load of the mother during pregnancy, maternal nutritional status during pregnancy, sex of the exposed infant, age of the infant during enrollment to PMTCT, CPT adherence of the infant, father's enrollment to ART, postnatal follow up, WHO clinical stage at baseline, and duration of breastfeeding were all associated with mother-to-child HIV transmission (Table 3).

In the multivariate logistic regression analysis, poor adherence of infant for CPT (AOR: 5.6; 95% CI: 1.010–27.24), father not enrolled to ART (AOR: 4.4; 95% CI: 1.187–15.724), age of infant at enrollment >6 weeks (AOR: 4.5; 95% CI: 1.102–16.1), mother's enrollment to PMTCT during labor and delivery or after (AOR: 6.84; 95% CI: 1.316–42.743), and mothers on the WHO clinical stage mild or advanced (AOR: 3.6; 95% CI: 1.146–16.842) were found to be the most important significant predictors of mother-to-child transmission of HIV (Table 4).

## 4. Discussion

Our study revealed that the cumulative incidence of MTCT of HIV among HIV-exposed infants in public general hospitals of the West Guji zone, Southern Ethiopia, is nearly 4%, which is a significant burden to our society.

The finding of our study was in line with the study conducted in Kenya in 2010 and Bahr Dar, Ethiopia in 2018 in which nearly 5% of the exposed infants were infected with HIV [15–17].

The result of this study was lower than the findings of retrospective studies conducted in Cameroon, Kenya, the Democratic Republic of Congo, Brazil, Uganda, India, South Gondar zone in Amhara Region, Dire Dawa Ethiopia, Gonder University Referral Hospital Northwest Ethiopia, East and West Gojjam Zones of Amhara Region, Southwest Ethiopia St. Mery Luke Referral Hospital, Asella Hospital Ethiopia [5, 18, 19] [16, 19–26]. This difference may be due to the reason that positive impact of B positive on the reduction of MTCT of HIV; the data of most of the studies were before the implementation of option B positive.

At present enrollment, time was the main predictor of MTCT of HIV, higher than the study conducted in Uganda where only 4% of the mothers of HEI were enrolled to ART before pregnancy compared to nearly 60% on HAART in our study. The other reason for this difference may be that most of the studies conducted before were in only one hospital [5, 13, 16, 19–24]. The other reason may also be that enrollment of the HIV positive mothers to ART and to PMTCT was significantly high in our findings compared to the other studies, 72.1% in Kenya and only 4% of mothers were on HAART before pregnancy in the study conducted in Uganda. The ANC follow-up coverage of HIV-positive pregnant mothers was also good compared with the studies before. Additionally, above 90% of the mothers were delivered at health institutions in our study; this was helpful to enroll the HEIs into the PMTCT service before six weeks of age for PMTCT of HIV [16, 19, 23–25]. In our study, none of the HIV-exposed infants were on mixed feeding practice, as mixed feeding practice before six months of age may cause infection of the gastrointestinal system which increases the risk of transmission of HIV.

This result was higher compared with the prospective study conducted in Nigeria in 2015 and a cross-sectional study conducted in the Tigray region in 2016 that found the rates of MTCT of HIV were 1% and 2.1%, respectively [23–25]. This difference may be due to study design differences, and less sample size and short duration of the study compared to the present.

In this study, in multivariate logistic regression, those HEI enrolled to PMTCT at the age of >6 weeks were significantly at risk to be HIV positive than their counterparts. This result was also supported by the study conducted in Gonder, Uganda [13, 24].

HEI whose mothers were enrolled in PMTCT followed up during labor and delivery or after are also more likely to be HIV positive than those whose mothers were on HAART before pregnancy. This result was supported by the study conducted in East and West Gojjam Zones, and retrospective follow-up studies conducted in Gonder, Ethiopia, and Cameroon [5, 13, 16, 27]. This is due to the reason that early detection and initiation of ARV drugs for HIV positive mothers can significantly reduce the chance of the child becoming infected with HIV [2].

HEI who had poor adherence for CPT at the end of follow-up were also at high likely to be HIV positive than those with good adherence. The reason for the HEI to have poor adherence may be poor communication and inadequate counseling for the mothers; they may not be motivated to take their child for follow-up and to take the medication and care services.

HEIs born from mothers with an advanced WHO clinical stage at baseline were also five times more likely to be HIV positive than those born from mothers with mild clinical stages at baseline. This finding was also supported by a study conducted in southwest Ethiopia and a case-control study in Addis Ababa [20, 28]. This may be due to the reason that HIV-positive mothers with advanced WHO clinical stages may have impairment of the placental barrier, may have a high viral load and different opportunistic infections, which may increase the risk of MTCT of HIV.

On the other hand, the independent variables infant birth weight <2.5 kg, maternal nutrition, time of knowing HIV status of the mother, viral load of the mother, maternal age, and residence were significantly associated with MTCT of HIV in all other studies done in Ethiopia, but in our study, these variables were not significantly associated.

## 5. Conclusion

The finding of our study revealed that the cumulative incidence of mother-to-child transmission of HIV among HIV-exposed infants in general hospitals of the West Guji Zone, Southern Ethiopia regional state was significantly high. Though this result shows positive in the elimination of mother-to-child transmission of HIV among HIV-exposed infants, there is still more awareness needed to make the MTCT of HIV 0%. The limitation of the study areas is that as retrospective study secondary data is used of HIV-exposed infants, there were 8.4% incomplete charts, some details could have been missed related to HIV-infected mothers who were not tested for HIV and those HIV-exposed infants who were not registered to PMTCT follow-up. However, the incidence of MTCT of HIV could be higher among those HIV-exposed infants not registered in the PMTCT follow-up and their mothers who have never even accessed health services and HIV testing during pregnancy or delivery. In addition, researchers recommend that health professionals working in health facilities should give due attention to detecting and enrolling those HIV positive mothers and their HIV positive partners in ART as early as possible and should focus on adherence counseling of mothers to have seriousness towards HEIs for CPT and to enroll those HEIs to PMTCT follow-up before six weeks of age. Furthermore, they should also link with the health extension workers to follow their adherence strictly.

## Figures and Tables

**Table 1:** Clinical characteristics of mothers of HIV-exposed infants in general hospitals of West Guji Zone, Oromia, Ethiopia, 2020 (*n* = 422).

Variables		Frequency	% (*n* = 422)	HIV test results of infants	
				Positive (%)	Negative (%)
The WHO clinical stage at baseline	Mild stage (I, II)	350	83	10 (2.0)	304 (72.0)
	Advanced stage (III, IV)	72	17	10 (2.0)	98 (23.0)
The WHO clinical stage at last visit of pregnancy	Mild stage (I, II)	400	95	3 (1.0)	398 (94.0)
	Advanced stage (III, IV)	22	5	7 (2.0)	14 (3.0)
CD4 count at baseline	≥500	72	17	0 (0.0)	48 (11.0)
	351–499	89	21	0 (0.0)	112 (27.0)
	200–350	175	41	3 (1.0)	176 (42.0)
	<200	86	20	10 (2.0)	73 (17.0)
CD4 count at pregnancy (*n* = 422)	≥500	166	39	1 (0.0)	186 (44.0)
	351–499	126	30	0 (0.0)	122 (29.0)
	200–350	86	20	3 (1.0)	83 (20.0)
	<200	44	10	8 (2.0)	19 (5.0)
Functional status at baseline	Working	298	71	7 (2.0)	248 (59.0)
	Ambulatory	113	27	7 (2.0)	109 (26.0)
	Bedridden	11	3	7 (2.0)	44 (10.0)
Time of knowing HIV status	Before pregnancy	294	70	9 (2.0)	277 (66.0)
	During pregnancy or after	128	30	5 (1.0)	131 (31.0)
Enrolment to PMTCT	Yes	396	94	14 (3.0)	312 (74.0)
	No	26	6	14 (3.0)	82 (19.0)
Time of enrolment to PMTCT	Before pregnancy on HAART	272	64	5 (1.0)	188 (45.0)
	During pregnancy	118	28	5 (1.0)	142 (34.0)
	During labor and delivery or after	32	8	5 (1.0)	77 (18.0)
ART adherence at last visit of pregnancy	Good	268	64	7 (2.0)	294 (70.0)
	Fair	86	20	3 (1.0)	68 (16.0)
	Poor	68	16	3 (1.0)	47 (11.0)
OIs at base line	No	182	43	2 (0.0)	211 (50.0)
	Yes	240	57	12 (3.0)	197 (47.0)
Disclosure status	Yes	316	75	7 (2.0)	322 (76.0)
	No	106	25	7 (2.0)	86 (20.0)
Last viral load during pregnancy	Undetectable	324	77	8 (2.0)	329 (78.0)
	Detectable	98	23	6 (1.0)	79 (19.0)
Nutritional status during pregnancy	Not malnourished	335	79	7 (2.0)	322 (76.0)
	Malnourished	87	21	6 (1.0)	89 (21.0)

**Table 2:** Obstetric characteristics and HIV-exposed infant characteristics in general hospitals of West Guji Zone, Oromia, Ethiopia, 2020 (*n* = 422).

Variables		Frequency	% (*n* = 422)	Last HIV test results of infants	
				Positive (%)	Negative (%)
ANC follow-up	Yes	395	94	13 (3.0)	381 (90.0)
	No	27	6	3(1.0)	24 (6.0)
Number of ANC visit (*n* = 422)	≥4	267	63	2 (1.0)	265 (63.0)
	3	123	29	9 (2.0)	114 (27.0)
	1	3	1	2 (2.0)	2 (1.0)
Postnatal follow-up	Yes	270	64	9 (2.0)	260 (62.0)
	No	152	36	7 (2.0)	146 (35.0)
Gravidity	I	67	16	2 (1.0)	66 (16.0)
	II	160	38	6 (1.0)	155 (37.0)
	III and above	194	46	8 (2.0)	185 (44.0)
Number of children (parity)	1	86	20	2 (1.0)	84 (20.0)
	2	160	38	9 (2.0)	151 (36.0)
	≥3	176	42	5 (1.0)	171 (40.0)
Place of delivery	Health institution	412	98	14 (3.0)	398 (94.0)
	Home	12	3	3 (1.0)	9 (2.0)
Mode of delivery	SVD	346	82	14 (3.0)	332 (79.0)
	CS	77	18	2 (1.0)	74 (18.0)
Sex of infant	Male	226	53	8 (2.0)	216 (51.0)
	Female	198	47	8 (2.0)	189 (45.0)
Birth weight in KG	≥2.5	391	93	14 (3.0)	378 (90.0)
	<2.5	33	8	3 (1.0)	30 (7.0)
Growth pattern	Normal	379	90	3 (1.0)	375 (89.0)
	Growth failure	44	10	13 (3.0)	31 (7.0)
Feeding practice before six months age	EBF	413	98	1 (0.0)	398 (4.0)
	ERF	10	2	1 (0.0)	10 (2.0)
Feeding practice after six months	BF + CF	400	95	14 (3.0)	385 (91.0)
	Stop BF	24	6	2 (1.0)	22 (5.0)
Duration of BF in weeks	≤ 48 weeks	199	47	10 (2.0)	192 (45.0)
	>48 weeks	226	53	10 (2.0)	214 (51.0)
ARV prophylaxis at birth for six weeks	Yes	398	94	3 (1.0)	394 (93.0)
	No	24	6	12 (3.0)	13 (3.0)
Age in weeks at enrolment to PMTCT	≤6	345	82	5 (1.0)	338 (80.0)
	>6	78	18	9 (2.0)	69 (16.0)
Adherence of infant	Good	320	76	7 (2.0)	29 (7.0)
	Fair	78	18	5 (1.0)	47 (11.0)
	Poor	24	6	5 (1.0)	35 (8.0)
Father's ART enrolment(*n* = 422)	Enrolled	259	61	6 (1.0)	251 (60.0)
	Not enrolled	45	11	10 (2.0)	38 (9.0)

**Table 3:** Bivariate analysis of predictors of MTCT of HIV in public general hospitals of West Guji Zone, Oromia, Ethiopia, 2020 (*n* = 422).

Variables		HIV test result of HEI		COR(95% CI)	*P* value
		Negative (%)	Positive (%)		
Residence	Urban	315	10	1	
	Rural	91	6	1.8 (0.67–5.21)	0.225
Occupation	Employed	216	6		
	Unemployed	189	10	0.763–5.783	0.151
Time of knowing HIV status of mother	Before pregnancy	278	10		
	During pregnancy or after	128	6	1.2 (0.42–3.25)	0.75
Time of enrolment to PMTCT	Before pregnancy on HAART	251	6	1	
	During pregnancy	120	5	1.7 (0.52–5.8)	0.365
	During labor and delivery or after	34	6	7.3 (2.24–23.9)	0.001
Disclosure status	Yes	310	8	1	
	No	95	8	3.6 (1.36–9.65)	0.01
Last viral load during pregnancy	Undetectable	329	9	1	
	Detectable	77	7	2.9 (1.10–8.09)	0.031
Nutritional status during pregnancy	Not malnourished	328	8	1	
	Malnourished	79	7	3.7 (1.38–9.83)	0.009
Sex of HEI	Male	216	8	1	
	Female	188	8	1.01 (.38–2.69)	0.971
Age of infant in weeks at enrolment to PMTCT	≤6	338	7	1	
	>6	69	9	7 (2.59–19.1)	0
Adherence of infant for PMTCT drugs	Good	313	6	1	
	Fair	73	6	3 (0.94–9.87)	0.063
	Poor	20	5	11 (3.27–8.26)	0
Father's enrolment to ART	Enrolled	251	7	1	
	Not enrolled	37	7	6.6 (2.34–18.57)	0
Postnatal follow-up	Yes	259	9	1	
	No	146	6	.463–3.326	0.668
The WHO stage of mother at baseline	Mild stage	331	7	1	
	Advanced stage	74	8	4.9 (1.85–13.24)	0.001

**Table 4:** Multivariate analysis of predictors of MTCT of HIV in public general hospitals of West Guji Zone, Oromia, Ethiopia, 2020 (*n* = 422).

Variables		COR (95% CI)	*P* value	AOR (95% CI)	*P* value
Residence	Urban			1	
	Rural	2.3 (0.67–5.21)	0.225	1.9 (.455–7.921)	.380
Occupation	Employed			1	
	Unemployed	2.7 (0.76–5.78)	0.151	.7 (.17–2.95)	0.655
Time of enrolment to PMTCT	Before pregnancy on HAART			1	
	During pregnancy	2.2 (0.52–5.8)	.365	4 (0.828–20.129)	.084
	During L&D or after	9.3 (2.24–23.9)	0.001	6.84 (1.316–42.743)	**.018**
Disclosure status	Yes			1	
	No	4.6 (1.36–9.65)	0.010	3.2 (0.886–12.198)	0.075
Last viral load during pregnancy	Undetectable			1	
	Detectable	3.7 (1.10–8.09)	0.031	1.07 (.236–4.868)	0.928
Nutritional status during pregnancy	Not malnourished			1	
	Malnourished	4.7 (1.38–9.83)	0.009	1.6 (.406–6.583)	0.490
Age of infant in weeks at enrolment	≤6 weeks			1	
	>6 weeks	8.9 (2.59–19.1)	0.001	4.5 (1.102–16.1)	**0.029**
Adherence of infant for PMTCT	Good			1	
	Fair	3.8 (0.94–9.87)	.063	3.9 (.860–17.58)	0.078
	Poor	14.0 (3.27–38.26)	0.001	5.4 (1.010–27.24)	**0.047**
Father's enrolment to ART	Yes			1	
	No	8.4 (2.34–18.57)	0.001	4.5 (1.187–15.724)	**0.026**
The WHO clinical stage of mother at baseline	Mild stage				
	Advanced stage	6.2 (1.85–13.24)	0.001	3.6 (1.146–16.842)	0.047

## Data Availability

Data utilized and assessed in the present study are available from the corresponding author on request.

## Abbreviations

ANC: Antenatal care
ART: Antiretroviral therapy
ARV: Antiretroviral
EBF: Exclusive breast feeding
EDHS: Ethiopian demographic and health survey
HIV: Human immunodeficiency virus
HIV-E: HIV exposed
PMTCT: Prevention of mother-to-child transmission.
